# AMIC achieves sustained clinical improvement in isolated patellar cartilage defects over 5 years, correlating with MRI

**DOI:** 10.1002/ksa.12518

**Published:** 2024-10-28

**Authors:** Nayana Joshi Jubert, Mercè Reverté Vinaixa, Irene Portas Torres, Daniel Moreno Martínez, Marcelo Casaccia, Marc Aguilar Garcia, Joan Pijoan Bueno, Enric Castellet Feliu, Joan Minguell Monyart

**Affiliations:** ^1^ Universitat Autònoma de Barcelona Departament de Cirurgia Bellaterra Spain; ^2^ Hospital Universitari Vall d'Hebron, Departament de Cirurgia Ortopèdica i Traumatologia, Unitat de Genoll Barcelona Spain; ^3^ Vall d'Hebron Institut de Recerca, Musculoskeletal Tissue Engineering group Barcelona Spain; ^4^ Institut de Diagnòstic per la Imatge Hospital Universitari Vall d'Hebron Barcelona Spain

**Keywords:** autologous matrix‐induced chondrogenesis, cartilage, chondral lesion, magnetic resonance imaging, patella

## Abstract

**Purpose:**

To evaluate 5‐year postoperative clinical outcomes of autologous matrix‐induced chondrogenesis (AMIC) for isolated ICRS grade 3–4 patellar cartilage defects and correlate outcomes with magnetic resonance imaging (MRI). The hypothesis was that AMIC would improve clinical symptoms and induce neocartilage formation, visible on MRI, making it a safe and effective option for repairing focal patellar cartilage defects.

**Methods:**

The cohort comprised 13 focal patellar lesions in 12 patients. Pain visual analogue scale (VAS), Knee Injury and Osteoarthritis Outcome Score (KOOS), Kujala score, EuroQol‐5D Health Survey questionnaire and MRI data were assessed preoperatively and at 2 and 5 years postoperatively. All MRI scans were evaluated using the Magnetic Resonance Observation of Cartilage Repair Tissue System. Descriptive statistics were calculated on all data. Inferential analysis comparing outcome scores before and after surgery employed the nonparametric Wilcoxon signed‐rank test, with the nonparametric Friedman test used to detect differences across multiple test attempts. *p* < 0.05 was considered statistically significant.

**Results:**

Twelve patients (23–52 years old) with patellofemoral chondral full‐thickness defects (2–4 cm^2^) were treated. At a 5‐year follow‐up, eleven knees showed MRI improvement. Two were asymptomatic and nine showed clear clinical improvement. Only one knee showed no clinical improvement. MRI revealed a defect filling with newly formed cartilage characterized by a less compact and heterogeneous signal. Cartilage degradation or joint damage was observed in two knees, and bone formation within the plate was identified in four. AMIC significantly improved patients' VAS pain, KOOS, EuroQol‐5D and Kujala scores compared to preoperative baseline for up to 5 years postoperatively.

**Conclusions:**

Satisfactory clinical outcomes and new cartilage formation, as observed by MRI, are achieved with AMIC at mid‐term follow‐up for ICRS grade 3–4 in small‐to‐medium‐sized patellar defects in patients under 52 years of age, with improvements maintained for up to 5 years.

**Level of Evidence:**

Level III.

## Graphical abstract

Autologous matrix‐induced chondrogenesis achieves clinical and magnetic resonance imaging improvement over 5‐year patellar cartilage defects.

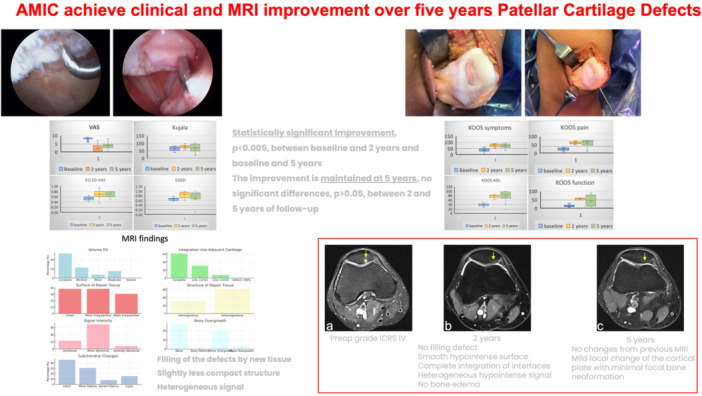

**Abbreviations**
ADL: activity daily living
AMIC: autologous matrix‐induced chondrogenesis
EQ‐5D: EuroQol‐5D
ICRS: International Cartilage Regeneration Joint Preservation Society
IKDC: International Knee Documentation Committee
KOOS: Knee Injury and Osteoarthritis Outcome Score
MOCART: magnetic resonance observation of cartilage repair tissue
MRI: magnetic resonance imaging
MSCs: mesenchymal stem cells
OATS: osteochondral autograft transfer system
QoL: quality of life
VAS: visual analogue scale

## INTRODUCTION

The management of symptomatic patellar chondral lesions remains controversial despite their high prevalence [39]. Surgical debridement and repair are the most common treatments but may be associated with a high risk of recurrence. Several techniques have been described to treat these injuries [17, 19, 36], ranging from the creation of simple microfractures to autologous chondrocyte implantation, osteochondral auto‐ or allograft or synthetic transplantation and stem cell therapy. However, these techniques have shown lower success rates for patellar cartilage defects than for femoral condyles [1, 15, 32], possibly due to the thicker patellar cartilage [5]. Acellular scaffolding, specifically a type I/III collagen membrane, has recently gained interest as a way to treat focal chondral defects since it can improve results, increase efficiency and simplify the surgical technique of existing procedures [16, 18, 26, 28, 38]. The autologous matrix‐induced chondrogenesis (AMIC) technique, which uses the type I/III collagen membrane to enhance microfractures, has shown promising results in the treatment of focal patellar cartilage injuries [1, 4, 8, 12, 15, 25, 35, 38].

This study is needed due to limited long‐term data on AMIC for isolated patellar chondral lesions, particularly in middle‐aged patients. Focusing solely on patellar defects provides new insights into AMIC's safety and efficacy based on 5‐year clinical and magnetic resonance imaging (MRI) outcomes. It can be clinically useful by showing that AMIC provides a cost‐effective, single‐surgery solution that promotes reliable cartilage regeneration, supporting its use for treating isolated patellar defects. The aim of this single‐cohort study was to evaluate clinical outcomes and correlate them with MRI at 5‐year follow‐up in patients over 18 years old with isolated patellar chondral lesions treated with the AMIC technique. The hypothesis was that AMIC would improve clinical symptoms and induce neocartilage formation, visible on MRI, making it a safe and effective option for repairing focal patellar cartilage defects.

## MATERIALS AND METHODS

The study was approved by the Hospital Universitari Vall d'Hebron Clinical Research Ethics Committee (PR(ATR)117/2016), and all enroled patients provided written informed consent. From January 2014 to February 2017, all patients presenting with an isolated ICRS grade 3–4 patellofemoral chondral defect as well as with persistent anterior knee pain and swelling after 6 months of conservative treatment were consecutively enroled in this observational case series. These patients were selected for AMIC treatment using a collagen matrix (porcine collagen type I/III, Chondro‐Gide®, Geistlich) and fibrin sealant (Tisseel Lyo; Baxter Healthcare Corp.). None of the patients had previously undergone surgery. None of the patients reported a traumatic event but rather chronic progressive pain. For this reason, conservative treatment was given prior to surgery.

Clinical outcomes were evaluated using the following assessment tools: (i) a visual analogue scale (VAS) to rate each patient's knee pain; (ii) Knee Injury and Osteoarthritis Outcome Score (KOOS) to assess both the short‐term and longer‐term consequences of knee injury, covering five domains (pain, other symptoms, function in activity daily living, function in sports and recreation and knee‐related quality of life [QoL]); (iii) Kujala score to specifically assess patellofemoral pain and (iv) the EuroQol‐5D (EQ‐5D) tool to rate health‐related QoL. Data were collected prospectively, including preoperative assessments and evaluations at 2 and 5 years after AMIC implantation.

MRI was used both to detect patellar cartilage lesions and to monitor chondral regeneration. Evaluations were conducted preoperatively, and both 2 and 5 years post‐AMIC implantation, using axial and sagittal T1‐weighted sequences with fat saturation. The quality of the repaired cartilage was assessed using the MOCART (Magnetic Resonance Observation of Cartilage Repair Tissue) 2.0 Knee score [31]. All images were evaluated by the same radiologist, who specialized in musculoskeletal imaging.

Inclusion criteria were a symptomatic isolated patellar chondral defect, 1.50–3.50 cm², confirmed and measured by MRI. Exclusion criteria included patient age outside the range of 18–55 years, patellofemoral malalignment (>10° tilt on axial radiographs or dysplasia of the patellofemoral compartment), tibiofemoral malalignment (>10° angulation on standing anteroposterior radiographs), and the presence of concomitant femoral, tibial or trochlear chondral lesions or ligament or meniscal injuries.

### Surgical technique

Under regional or general anaesthesia with antibiotic prophylaxis, a thigh tourniquet was applied. The procedure can be performed without ischaemia. Arthroscopy was performed to assess the cartilage defect, recording its location, size and depth, using the ICRS score. The damaged cartilage was debrided, loose chondral flaps and necrotic tissues were removed and the calcified chondral layer was cleared. Then, utilizing a mini‐open approach and 1 mm drill, perforations were created to a depth of 9 at 5 mm intervals. A clear border of normal adjacent cartilage was defined with a punch from the Arthrex osteochondral autograft transfer system reconstruction kit, and circular patches of the collagen matrix were applied in the defect area, overlapping as needed [29]. Successively, fibrin sealant was applied to cover all membrane areas, with the removal of any excess glue. The knee joint was held in an extended position for 5 min, ensuring appropriate adhesion and stability of the collagen matrix, as described elsewhere [12].

### Postoperative care

A standardized postoperative rehabilitation protocol was initiated 24 h postimplantation. For this, patients were referred to a rehabilitation unit where they were monitored closely by medical staff. All patients adhered to their prescribed physiotherapy programme. They were instructed to use crutches with weight‐bearing as tolerated (no braces used). The passive range of motion exercises began immediately using a continuous passive‐motion machine, starting from 0° to 30° for 2 weeks and increasing by 30° every 2 weeks. Static quadriceps exercises were incorporated to prevent adhesions and promote joint nutrition.

Unrestricted weight‐bearing and range of motion were encouraged after 6 weeks and cycling and swimming exercises also started to increase strength, as tolerated. Around 6 months, slow line running was permitted, with high‐impact activities allowed 12 months postimplantation.

### Statistical analysis

All data were analyzed using SPSS (Student Version‐16.0, SPSS Inc.). Descriptive statistics, including minimum and maximum values, arithmetic means, standard deviations and quartiles, were calculated for all continuous variables.

Inferential analysis to compare outcome scores before and after surgery was conducted using paired‐sample comparisons with the nonparametric Wilcoxon signed‐rank test. Additionally, the nonparametric Friedman test was employed to detect differences across multiple test attempts. Due to multiple comparisons and the small sample size, the *p* value criterion for statistical significance was adjusted by Bonferroni correction (*p *= alpha error [0.05] divided by the number of comparisons [2] = 0.025).

## RESULTS

**Figure 1:**
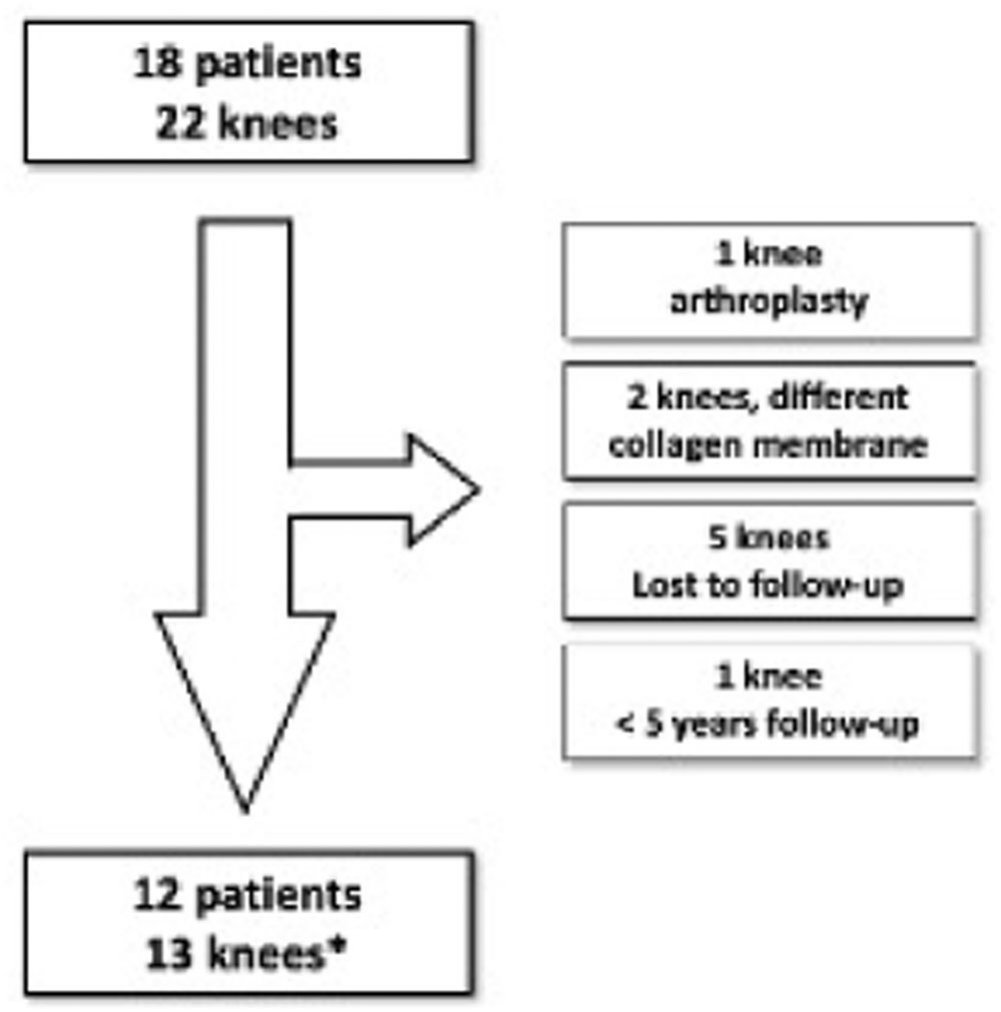
Patient enrolment flow chart. * One patient had an autologous matrix‐induced chondrogenesis procedure in both knees.

From 2014 to 2017, 18 consecutive patients (22 knees) were treated with the AMIC procedure for symptomatic isolated ICRS grade 3–4 patellar chondral defects. Nine of these procedures and six of these patients were excluded: one procedure involved unicompartmental knee arthroplasty due to a femoral condyle injury; two knees were treated using a different collagen I/III membrane; five cases were lost to follow‐up and one had follow‐up <5 years (Figure 1). Hence, 13 knees (12 patients) were included in the final analysis. All cases were nontraumatic, and no patient had a high‐demand activity level (practicing sports regularly). Four patients were considered nondemand activity patients (participating in sports only occasionally), while eight were considered low‐demand (sedentary, with <3 h of physical activity/day). No patient underwent additional surgical procedures over the study period. No adverse events related to implants or surgery, postoperative infections or other complications were observed. Summary characteristics of the overall cohort are summarized in Table 1.

**Table 1:** Cohort characteristics.

Age, years, median (SD)	40 (9.5)
Female sex, *n* (%)	7 (53.8)
Side, right/left, *n*	8/5
BMI, Kg/m^2^, median (SD)	26 (4.3)
Lesion size, cm^2^, mean ± SD (range)	2.6 ± 0.8 (2‐4)
Lesion location, *n* (%)	
Medial facet	1 (7.7)
Lateral facet	9 (69.2)
Central	3 (23.1)

Abbreviation: BMI, body mass index.

### Clinical outcomes

At 5‐year follow‐up, significant clinical improvement was observed, as summarized in Table 2. Dissatisfaction with the outcome was reported only by the one patient who had undergone the procedure in both knees (knees #3 and #10).

**Table 2:** Clinical outcomes at baseline, 2‐year and 5‐year follow‐up of the cohort.

	Baseline	2 years	5 years
VAS	8 ± 0.8 (6–9) 8 [8, 8.5]	2.4 ± 2.1 (0–7) 2 [0.5, 4]	3.8 ± 2.3 (0–8) 4 [2.5, 5]
KOOS symptoms	35.7 ± 15.8 (0–60.7) 35.7 [26.8, 44.6]	75 ± 11.4 (53.6–89.3) 78.6 [66, 85.7]	72.8 ± 19.5 (32.1–100) 75 [58.9, 85.7]
KOOS pain	29.7 ± 9.6 (11.1–41.6) 30.6 [20.8, 38.9]	78.4 ± 8.3 (63.9–91.7) 77.8 [72.2, 86.1]	77.1 ± 24.2 (16.7–100) 86.1 [61.1, 94.4]
KOOS ADL	39.1 ± 6.4 (27.9–48.5) 41.2 [33.8, 43.4]	76.8 ± 7.7(63.2–85.3) 79.41 [71.32, 83.09]	77.9 ± 24,9 (19.1– 100) 85.3 [67.6, 97.8]
KOOS SP	16.1 ± 6.8 (5–30) 15 [10, 20]	55 ± 4.6 (50–60) 55 [50, 60]	44.2 ± 31.8 (0–90) 45 [12.5, 77.5]
KOOS QoL	16.3 ± 7.5 (0–31.2) 18.7 [12.5, 18.7]	59.6 ± 14.7 (25–81.2) 62.5 [50, 68.7]	49.5 ± 30.8 (0–100) 56.2 [21.9, 71.9]
Kujala	64 ± 15.1 (38–81) 67 [53, 77.5]	76.6 ± 11.4 (57–98) 76 [69.5, 84]	67 ± 26.6 (17–99) 70 [46, 94]
EQ‐5D	0.7 ± 0.1 (0.6–0.8) 0.7 [0.6, 0.7]	0.8 ± 0.1 (0.6–1) 0.9 [0.7, 0.9]	0.7 ± 0.3 (0.1–1) 0.8 [0.6, 0.9]
EQ‐5D VAS	0.5 ± 0.1 (0.4–0.6) 0.5 [0.5, 0.6]	0.7 ± 0.2 (0.3–0.9) 0.7 [0.6, 0.8]	0.7 + 0.1 (0.5–0.8) 0.8 [0.6, 0.8]

*Note*: Data are provided as mean ± SD (range) and median (25th percentile, 75th percentile).

Abbreviations: ADL, activity daily living; EQ‐5D, EuroQol‐5D; KOOS, Knee Injury and Osteoarthritis Outcome Score; QoL, quality of life; VAS, visual analogue scale.

The VAS pain score decreased significantly from baseline to both 2‐ and 5‐year follow‐up (*p* < 0.005). At 5 years, the average VAS pain rating was slightly higher than at the 2‐year assessment (by 1.5 points; *p* = 0.002) but it remained significantly lower than at the presurgery baseline (reduced from 8 to 3.8, *p* < 0.001). At the 2‐year follow‐up, VAS pain scores were >4 in 2 patients with cartilage defect filling scores of 5 and 15. At the 5‐year follow‐up, VAS scores ≥4 were observed in five patients with the following cartilage defect filling scores: two patients scored 5 points, two patients scored 15 points and one patient scored 20 points.

All KOOS subdomains improved postoperatively, with statistically significant differences (*p* = 0.001) between baseline and both the 2‐ and 5‐year follow‐up assessments. Most of the KOOS subscales showed a slight worsening from 2 to 5 years, but none of these decreases were statistically significant (n.s.).

The Kujala score improved in all but one patient: the patient with bilateral knee procedures (knees #3 and #10). No significant differences (n.s.) were noted overall between the baseline Kujala score and either the 2‐ or 5‐year follow‐up assessments or between the 2‐ and 5‐year follow‐up evaluations (n.s.). Statistically significant differences were found between the baseline and 2‐year follow‐up (*p* = 0.015), indicating substantial functional improvement postoperatively.

Statistically significant differences were found between the baseline and 2‐year follow‐up (*p* = 0.015), indicating substantial functional improvement postoperatively. No significant differences (n.s.) were noted between the 2‐ and 5‐year follow‐up evaluations.

The EQ‐5D score also improved for all patients at 2‐year follow‐up versus baseline (*p* = 0.001). Although the average EQ‐5D score worsened slightly between the 2‐ and 5‐year assessments, again no statistically significant difference was found (n.s.), though the score at 5 years of follow‐up was no longer significantly higher than at the baseline. On the other hand, the EQ‐5D VAS pain rating improved significantly from baseline to both 2‐ and 5‐year follow‐up (*p* = 0.002), again with no statistically significant change between 2 and 5 years (n.s.).

### MRI

**Figure 2:**
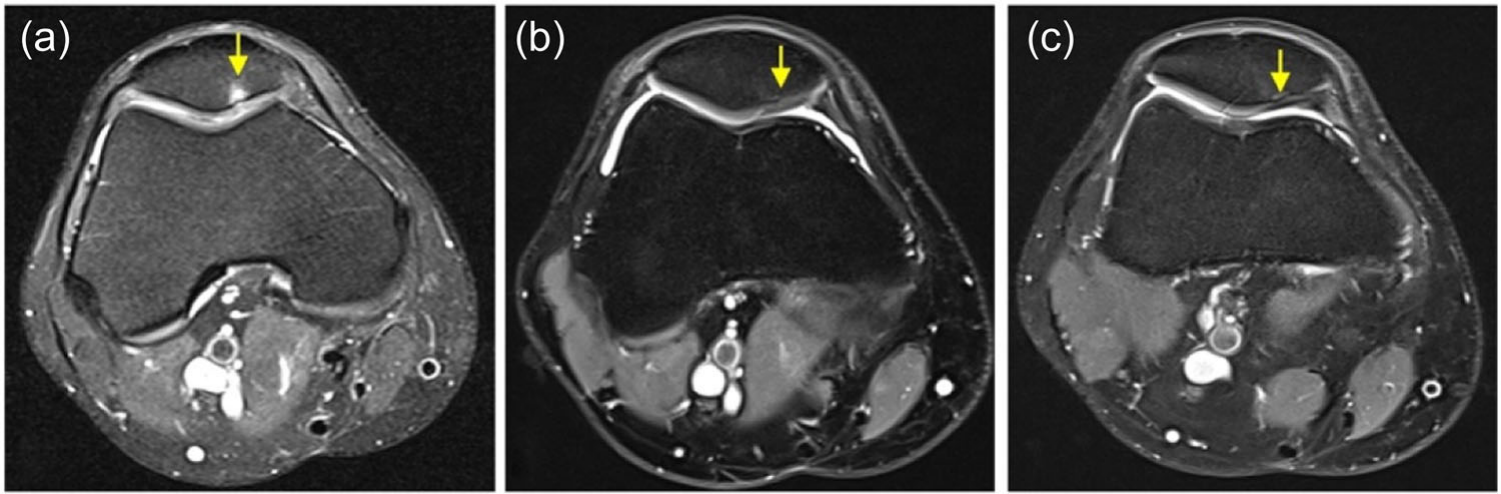
Example of good tissue regeneration, the magnetic resonance imaging (MRI) of patient #1, right knee. (a) Preoperative MRI: Focal chondral lesion of the patella grade International Cartilage Regeneration Joint Preservation Society IV located in the medial facet (arrow). (b) MRI at 2 years: No filling defect, smooth hypointense surface, complete integration of interfaces, heterogeneous hypointense signal and no bone oedema (arrow). (c) MRI at 5 years: No changes from previous MRI except for the presence of a mild focal change of the cortical plate with minimal focal bone neoformation (arrow).

**Figure 3:**
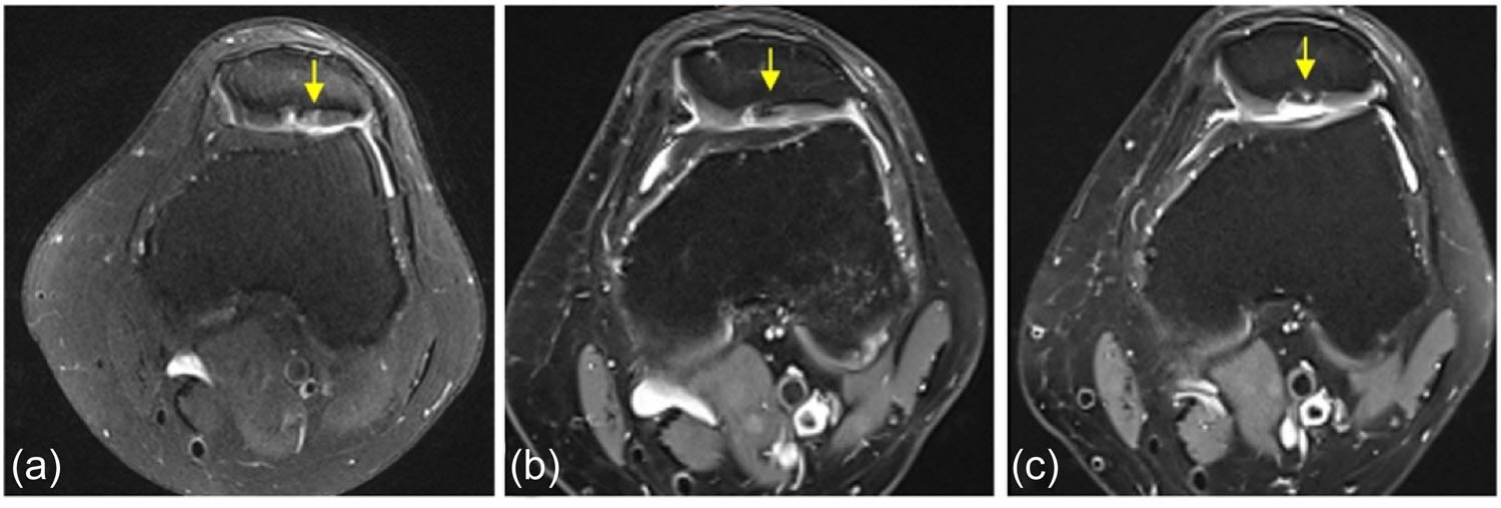
Example of poor tissue regeneration, the magnetic resonance imaging (MRI) of patient #12, left knee. (a) Preoperative MRI: Focal chondral lesion of the patella grade International Cartilage Regeneration Joint Preservation Society IV located in the central‐lateral facet (arrow). (b) MRI at 2 years: Decrease of the new tissue thickness, no significant signs of delamination, presence of a subchondral osteophyte, smooth interface edges and a small subcortical cyst in the plate (arrow). (c) MRI at 5 years: No changes from the previous MRI except for subchondral osteophyte growth (arrow).

Overall, the final MRI evaluation revealed the filling of the defects by new tissues, with a slightly less compact structure than native cartilage and a heterogeneous signal. Cartilage degradation or joint damage was observed in two knees, and bone formation within the plate was identified in four. Examples of MRI are shown in Figures 2 and 3.

Table 3 summarizes the MRI findings at the 5‐year follow‐up according to the parameters studied. Regarding volume filling, a comparison of 2‐year and 5‐year MRIs showed stable cartilage repair in most patients, with one knee improving and another deteriorating moderately.

**Table 3:** MRI findings at the 5‐year follow‐up.

Volume fill of cartilage defect^a^	Complete 7 (53.9)	Minimal defect 3 (23.1)	Minor defect 1 (7.7)	Moderate defect 2 (15.4)	Sever defect 0 (0)
Integration into adjacent cartilage^b^	Complete 8 (61.5)	Line <2 mm 4 (30.8)	Line >2 mm 1 (7.7)	Defect >50% 0 (0)	
Surface of the repair tissue^c^	Intact 5 (38.5)	Minor 5 (38.5)	Major 3 (30.8)		
Structure of the repair tissue (signal)	Homogeneous 4 (30.8)	Inhomogeneous 8 (61.5)			
Signal intensity of the repair tissue	Isointense 3 (23.1)	Minor abnormal 9 (69.2)	Severely abnormal 1 (7.7)		
Bony defect or bony overgrowth^d^	None 7 (53.8)	Bony defects 0 (0)	Minor overgrowth 5 (38.5)	Major overgrowth 1 (7.7)	
Subchondral changes^e^	Intact 6 (46.2)	Minor oedema 4 (30.8)	Severe oedema 1 (7.7)	Cysts 2 (15.4)	

*Note*: Data are provided as *n* (%).

Abbreviation: MRI, magnetic resonance imaging.

^a^ Complete (100% filling), minimal (1%–25% underfilling of the defect), minor (26%–50% underfilling of the defect), moderate (51%–75% underfilling of the defect), severe (>75% underfilling of the defect).

^b^ Complete (imperceptible interface between the repair tissue and the adjacent cartilage), line < 2 mm (split‐like demarcation line, measuring <2 mm between the repair tissue and the adjacent cartilage), line > 2 mm (split‐like demarcation line, measuring >2 mm between the repair tissue and the adjacent cartilage but <50% of repaired tissue length), defects ≥50% (≥50% of the repaired tissue length).

^c^ Intact (repair tissue surface is preserved and congruent, independent of the volume of filling of the cartilage defect), Minor Irregularities (<50% of the total repaired tissue diameter), Major Irregularities (>50% of the total repaired tissue diameter).

^d^ None (intact subchondral bone and the absence of intrachondral osteophytes), Minor overgrowth (bony overgrowth covering <50% of the adjacent native cartilage thickness), Major overgrowth (bony overgrowth covering >50% of the adjacent native cartilage thickness).

^e^ Minor (oedema‐like marrow signal affecting <50% of the repair tissue diameter), Severe (oedema‐like marrow signal impacting >50% of the repair tissue diameter), Cysts (subchondral cysts with a diameter ≥5 mm).

## DISCUSSION

The most important finding was the sustained and significant clinical improvement observed for up to 5 years after implantation, with no significant changes noted between the two‐ and 5‐year assessments. Clinical outcomes at 2 years postoperatively were sustained at the 5‐year mark, consistent with findings in published series demonstrating improvement across all clinical categories at 2 and 5 years [12, 25, 34, 35]. Notably, all three clinical scores (VAS, KOOS, Kujala) improved from baseline through 5 years. Although there was a slight decrease in some clinical scores at 5 years, the differences were not statistically significant, and improvements from baseline persisted. QoL improvements, assessed using EQ‐5D and KOOS‐QoL, were maintained at 5 years, indicating long‐term benefits.

While sustained clinical improvement beyond 5 years could not be confirmed, other studies have reported maintained improvements over 7 years following AMIC. However, many of these studies included various anatomical locations (patellar, trochlear, condylar and tibial defects) and did not distinguish between primary and revision settings [2, 3, 5, 14, 18, 20, 25, 28, 34, 37]. Recent research focusing on patellar defects shows promising results with significant functional and clinical score improvements lasting at least 5 years [12, 35, 38]. It is important to note that, in some of the latter series [38], most patients underwent corrective surgery for patellar instability (93.5%), which may have positively biased the overall results. This is in line with published data reflecting favourable outcomes up to 9 years when any malalignment is corrected [20].

Recent systematic reviews, meta‐analyses and randomized controlled trials (RCTs) support the effectiveness of AMIC in treating focal chondral defects in the knee, showing stable clinical outcomes and better scores compared to microfracture alone [11, 22, 23, 25, 34, 37]. AMIC has demonstrated superior outcomes for small‐ to medium‐sized defects in mid‐ to long‐term follow‐ups. Most of these studies found reduced VAS pain scores and improved knee scores in the first 2 years after using AMIC to treat focal chondral defects in the knee, with improvements maintained for up to 5 years [11, 34]. Additionally, AMIC shows comparable results to other surgical strategies for chondral regeneration in small‐ to medium‐sized cartilage defects [18, 21, 22]. In a Bayesian network meta‐analysis of clinical trials comparing surgical interventions for knee chondral defects, the AMIC procedure demonstrated superior overall outcomes to other approaches at approximately 3 years of follow‐up [26].

Significant improvements in clinical outcomes for AMIC‐treated patients within the first 2 years were demonstrated in the study (assessed using four different scales), with this improvement maintained through 5 years, generally agreeing with the literature [18].

Additional considerations include evaluating the influence of chondral lesion characteristics on final clinical outcomes. Defect size was not analyzed due to the small cohort, but existing studies suggest that outcomes are impacted by size, with worse results observed for larger defects [16, 27]. Defects were limited from 1.5 to 3.5 cm² by the inclusion criteria, which may explain the generally satisfactory outcomes. This criterion was implemented as the AMIC technique is generally not applied to large defects [18]. Nonetheless, choosing AMIC over microfracture alone, despite the latter being recommended for defects up to 2.5 cm^2^, might be justified by the high failure rates of microfracture alone when treating patellar lesions [23, 35]. Age, gender, body mass index and lesion location were also not analyzed due to the small cohort. Inconsistent findings have been reported in the literature regarding the effect of these characteristics on outcomes, not only for the AMIC technique but also for other techniques [2, 10, 12, 13, 16, 25, 27].

MRIs were performed at both two and 5 years post‐AMIC, as these milestones were considered crucial for assessing short‐ and mid‐term repair outcomes. However, the favourable clinical outcomes of AMIC were not confirmed by MRI findings regarding the homogeneity and quality of the newly formed tissue, although the overall MOCART score for each patient was found to be good at 2 years and maintained at 5 years of follow‐up. Some studies have already reported satisfactory clinical improvement 2 years after AMIC, but also a tendency toward repair tissue deterioration [8]. A similar trend was observed in this study at 2 years, with a tendency toward cartilage heterogeneity on MRI, but no increase in this heterogeneity was noted at 5 years of follow‐up. Therefore, it is suggested that failures at the 2‐year mark should be evaluated by clinical assessment rather than imaging. This radiological–clinical dissociation has been reported for other cartilage repair methods [28, 30, 34] and is assumed to be due to the long‐term nature of cartilage repair. Nevertheless, the quality of the morphological assessment using the MOCART score is controversial, and previous studies have shown its potential to predict clinical outcomes [7]. The defect fill rate appears to be a more reliable predictor of outcome than other MRI characteristics, for which limited or no correlations have been found.

The 5‐year follow‐up results found in this study align with previously published data [34], indicating that the majority of patients with satisfactory clinical outcomes experience complete filling of their cartilage defect. However, the final MRI evaluations revealed a somewhat less compact structure than native cartilage and a heterogeneous signal.

Similar findings have been described in other publications, including those using cells, even after 2 years of follow‐up [9, 18, 37].

Furthermore, complications like hypertrophy, osteophyte formation and bone marrow changes vary considerably depending on the technique used. For example, patients whose procedure involves a periosteum flap are more likely to develop hypertrophy and those treated with more aggressive microfractures are more likely to have subchondral bone involvement [8, 23]. To avoid complications involving the subchondral bone at the level of the patella, it is recommended that microfractures should not be performed. First, the surrounding healthy cartilage tissue is expected to gradually invade the membrane, and second, studies have shown that the contribution of autologous mesenchymal stem cells generated by microfractures is minimal [6].

Given that homogeneity was favoured when defining the cohort, the main limitation of this study is the small sample size. Statistically significant differences in MRI results between the 2‐year and 5‐year follow‐ups could not be detected, possibly due to underpowering from the small sample size or insufficient statistical power to reliably detect existing differences. Patients with acute traumatic injuries, concomitant knee injuries and previous surgery before cartilage repair were excluded to ensure a more homogeneous sample regarding the type of cartilage defect, as has been done by other authors [33]. However, such specificity poses recruitment challenges, which is why a multicentre study may be advantageous. Rejecting patients who require additional therapeutic procedures may introduce bias compared to the actual standard of care but helps to focus on AMIC outcomes. Larger series covering the major joints (hip, knee, talus) offer more patients but do not focus exclusively on the patella or knee [11, 18].

This study has additional limitations, including the lack of a control group and patient cohort heterogeneity, although this heterogeneity enhances external validity. While RCTs often focus on specific populations, such as young athletes, they may not capture the broader patient spectrum [34]. Therefore, this study provides valuable insights into real‐world outcomes across a more representative patient sample, even if precise subgroup analyses are limited.

Despite these limitations, the present study offers valuable insights into long‐term AMIC outcomes for patellar injuries, with strengths including a 100% follow‐up rate, the use of multiple validated clinical outcome scores (including the patella‐specific Kujala score) and the use of MRI to assess both preoperative cartilage status and postoperative newly formed tissues at two and 5 years post‐AMIC.

In addition, the average age of the sample (40 years) is a strength rather than a weakness of the study, as it is well established that chondral repair/regeneration is typically less favourable in patients over 30 years of age [16, 25, 34, 35]. Despite this, patients generally experienced improvements in both clinical and functional parameters, even though they were somewhat more advanced age.

The lack of consensus on the preferred treatment highlights the importance of patient and lesion characteristics, resource availability, cost and the surgeon's expertise in choosing the most appropriate method. For isolated patellar small to medium‐sized cartilage defects, AMIC is a potentially preferable option, as it avoids autologous cartilage harvesting, reduces costs and obviates the need for chondrocyte expansion in a separate laboratory.

Future research could explore the combination of the AMIC technique with allogeneic cells, such as those from the umbilical cord, to potentially enhance cartilage regeneration. This single surgical procedure may provide the advantages of reduced morbidity, lower cost and time efficiency compared to other techniques [24].

## CONCLUSIONS

Satisfactory clinical outcomes and new cartilage formation, as observed by MRI, are achieved with AMIC at mid‐term follow‐up for ICRS grade 3–4 in small‐to‐medium‐sized patellar defects in patients under 52 years of age, with improvements maintained for up to 5 years.

## AUTHOR CONTRIBUTIONS

Nayana Joshi Jubert contributed to conceptualization, data collection, formal analysis, methodology, project administration, resources, writing—original draft, writing—review and editing and supervision. Mercè Reverté Vinaixa contributed to statistical analysis, writing—review and editing. Irene Portas Torres contributed to data collection, writing—review and editing. Daniel Moreno Martínez contributed to radiological analysis. Marcelo Casaccia, Marc Aguilar Garcia, Joan Pijoan Bueno, Enric Castellet Feliu and Joan Minguell Monyart contributed to writing—review and editing.

## CONFLICT OF INTEREST STATEMENT

The authors declare no conflict of interest.

## ETHICS STATEMENT

Ethical approval for this study was obtained from the local ethical committee of Hospital Universitari Vall Hebron, Barcelona, Spain. All included patients gave their written informed consent.

## Supporting information

Supporting information.

## Data Availability

The data that support the findings of this study are available from the corresponding author (N. J. J.), upon reasonable request.
