# Supplementary material for: AMIC achieves sustained clinical improvement in isolated patellar cartilage defects over 5 years, correlating with MRI
Source: Knee Surg Sports Traumatol Arthrosc. 2024 Oct 28;33(6):2104–13. doi: 10.1002/ksa.12518 (PMC12104774; doi:10.1002/ksa.12518)
Supplement: Supplementary file 1 — Supporting information. [file KSA-33-2104-s001.docx]

**Table 1. Cohort characteristics**

| Age, years, median (SD) | | 40 (9.5) |
| --- | --- | --- |
| Female sex, n (%) | | 7 (53.8) |
| Side, right/left, n | | 8/5 |
| BMI, Kg/m2, median (SD) | | 26 (4.3) |
| Lesion size, cm2, mean ± SD (range) | | 2.6 ± 0.8 (2-4) |
| Lesion location, n (%) | |  |
|  | Medial facet | 1 (7.7) |
|  | Lateral facet | 9 (69.2) |
|  | Central | 3 (23.1) |

**Table 2. Clinical Outcomes at baseline, 2-years and 5-years follow-up of the cohort.**

|  | **Baseline** | **2 years** | **5 years** |
| --- | --- | --- | --- |
| **VAS** | 8 + 0.8 (6 to 9)  8 [8, 8.5] | 2.4 + 2.1 (0 to 7)  2 [0.5, 4] | 3.8 + 2.3 (0 to 8)  4 [2.5, 5] |
| **KOOS Symptoms** | 35.7 + 15.8 (0 to 60.7)  35.7 [26.8, 44.6] | 75 + 11.4 (53.6 to 89.3)  78.6 [66, 85.7] | 72.8 + 19.5 (32.1 to 100)  75 [58.9, 85.7] |
| **KOOS**  **Pain** | 29.7 + 9.6 (11.1 to 41.6)  30.6 [20.8, 38.9] | 78.4 + 8.3 (63.9 to 91.7)  77.8 [72.2, 86.1] | 77.1+ 24.2 (16.7 to 100)  86.1 [61.1, 94.4] |
| **KOOS**  **ADL** | 39.1 + 6.4 (27.9 to 48.5)  41.2 [33.8, 43.4] | 76.8 + 7.7(63.2 to 85.3)  79.41 [71.32, 83.09] | 77.9+ 24,9 (19.1 to 100)  85.3 [67.6, 97.8] |
| **KOOS**  **SP** | 16.1 + 6.8 (5 to 30)  15 [10, 20] | 55 + 4.6 (50 to 60)  55 [50, 60] | 44.2 + 31.8 (0 to 90)  45 [12.5, 77.5] |
| **KOOS**  **QOL** | 16.3 + 7.5 (0 to 31.2)  18.7 [12.5, 18.7] | 59.6 + 14.7 (25 to 81.2)  62.5 [50, 68.7] | 49.5 + 30.8 (0 to 100)  56.2 [21.9, 71.9] |
| **Kujala** | 64 + 15.1 (38 to 81)  67 [53, 77.5] | 76.6 + 11.4 (57 to 98)  76 [69.5, 84] | 67 + 26.6 (17 to 99)  70 [46, 94] |
| **EQ-5D** | 0.7 + 0.1 (0.6 to 0.8)  0.7 [0.6, 0.7] | 0.8 + 0.1 (0.6 to 1)  0.9 [0.7, 0.9] | 0.7 + 0.3 (0.1 to 1)  0.8 [0.6, 0.9] |
| **EQ-5D VAS** | 0.5 + 0.1 (0.4 to 0.6)  0.5 [0.5, 0.6] | 0.7 + 0.2 (0.3 to 0.9)  0.7 [0.6, 0.8] | 0.7 + 0.1 (0.5 to 0.8)  0.8 [0.6, 0.8] |

Data are provided as mean ± SD (range) and median [25th percentile, 75th percentile]. VAS, visual analog scale. KOOS, Knee Injury and Osteoarthritis Outcome Score

**Table 3. MRI findings at the 5-year follow-up.**

| **Volume fill of cartilage defect**^1^ | Complete  7 (53.9) | Minimal defect  3 (23.1) | Minor defect  1 (7.7) | Moderate defect  2 (15.4) | Sever defect  0 (0) |
| --- | --- | --- | --- | --- | --- |
| **Integration into adjacent cartilage^2^** | Complete  8 (61.5) | Line <2mm  4 (30.8) | Line >2mm  1 (7.7) | Defect >50%  0 (0) |  |
| **Surface of the repair tissue^3^** | Intact  5 (38.5) | Minor  5 (38.5) | Major  3 (30.8) |  |  |
| **Structure of the repair tissue** (signal) | Homogeneous  4 (30.8) | Inhomogeneous  8 (61.5) |  |  |  |
| **Signal intensity of the repair tissue** | isointense  3 (23.1) | minor abnormal 9 (69.2) | severely abnormal  1 (7.7) |  |  |
| **Bony defect or bony overgrowth^4^** | None  7 (53.8) | bony defects  0 (0) | Minor overgrowth  5 (38.5) | Major overgrowth  1 (7.7) |  |
| **Subchondral changes^5^** | Intact  6 (46.2) | Minor edema  4 (30.8) | Severe edema  1 (7.7) | Cysts  2 (15.4) |  |

Data are provided as n (%)

^1^: Complete (100% filling), minimal (1-25% underfilling of the defect), minor (26-50% underfilling of the defect), moderate (51-75% underfilling of the defect), severe (>75% underfilling of the defect).

^2^: Complete (imperceptible interface between the repair tissue and the adjacent cartilage), line<2mm (split-like demarcation line, measuring <2mm between the repair tissue and the adjacent cartilage), line>2mm (split-like demarcation line, measuring >2mm between the repair tissue and the adjacent cartilage but <50% of repaired tissue length), defects ≥50% (≥50% of the repaired tissue length).

^3^: Intact (repair tissue surface is preserved and congruent, independent of the volume of filling of the cartilage defect), Minor Irregularities (<50% of the total repaired tissue diameter), Major Irregularities (>50% of the total repaired tissue diameter).

^4^: None (intact subchondral bone and the absence of intra-chondral osteophytes), Minor overgrowth (bony overgrowth covering <50% of the adjacent native cartilage thickness), Major overgrowth (bony overgrowth covering >50% of the adjacent native cartilage thickness).

^5^: Minor (edema-like marrow signal affecting <50% of the repair tissue diameter), Severe (edema-like marrow signal impacting >50% of the repair tissue diameter), Cysts (subchondral cysts with a diameter ≥5mm).
